# Enhancement of field-effect mobility due to structural ordering in poly(3-hexylthiophene) films by the dip-coating technique

**DOI:** 10.1107/S0021889813004718

**Published:** 2013-06-07

**Authors:** Kamran Ali, Ullrich Pietsch, Souren Grigorian

**Affiliations:** a Department of Physics, University of Siegen, Walter-Flex-Strasse 3, Siegen, 57072, Germany

**Keywords:** structural anisotropy, electrical anisotropy, grazing-incidence X-ray diffraction

## Abstract

Organic field-effect transistors (OFETs) were fabricated by depositing a regioregular poly(3-hexylthiophene) (P3HT) active layer using a dip-coating method. The field-effect mobility in OFETs depends on chain orientation and crystallinity and is related to direction and withdrawal speed with respect to the source/drain orientation. In this paper, how to control the structural and transport properties of P3HT films by coating parallel and perpendicular to the dipping direction is demonstrated. X-ray diffraction curves taken in the perpendicular direction exhibit a higher degree of crystalline ordering and edge-on conformation compared with those in the parallel direction; this finding correlates with the directional anisotropy of the OFET mobility. Both structural anisotropy and transport properties are enhanced upon thermal treatment.

## Abstract 2

Dip coating is a facile technique to control structure and electrical performance. Both structural anisotropy and transport properties are enhanced upon thermal treatment.

## 1. Introduction

Poly(3-hexylthiophene) (P3HT) is one of the promising π-conjugated polymers for organic field-effect transistors (OFETs) and organic solar cells because of its solution processability (Bao *et al.*, 1996 ▶; Joshi *et al.*, 2009 ▶; Surin *et al.*, 2006 ▶; Verilhac *et al.*, 2006 ▶; Yang *et al.*, 2005 ▶), high field-effect mobility (Kline *et al.*, 2005 ▶; Majewski *et al.*, 2006 ▶; Sirringhaus *et al.*, 1999 ▶; Surin *et al.*, 2006 ▶; Wang *et al.*, 2003 ▶) and low-cost fabrication (Karakawa *et al.*, 2008 ▶; Sirringhaus, 2005 ▶; Verilhac *et al.*, 2006 ▶). P3HT-based field-effect transistors are usually fabricated by spin coating (Aasmundtveit *et al.*, 2000 ▶; Joshi *et al.*, 2008 ▶; Surin *et al.*, 2006 ▶), drop casting (Shabi *et al.*, 2012 ▶) and inkjet printing techniques (Eom *et al.*, 2010 ▶).

In order to enhance structural properties, many research groups have examined different fabrication steps to improve chain orientation by self-assembly of the molecules in the liquid crystal phase, by substrate texturing and by the use of chemically patterned surfaces (De Luca *et al.*, 2011 ▶; Kim *et al.*, 2005 ▶; Xue *et al.*, 2010 ▶; Yasuda, 2010 ▶). The most extensively investigated factor among others affecting the field-effect mobility is the molecular ordering of nanocrystallites in P3HT thin-film transistors (Sirringhaus *et al.*, 1999 ▶; Cho *et al.*, 2006 ▶; Hao *et al.*, 2007 ▶; Joshi *et al.*, 2008 ▶). An alternative way to enhance the charge carrier transport is the dip-coating technique, which can facilitate self-assembly into a nanofibrillar lamellar structure (Sirringhaus *et al.*, 1999 ▶; Salleo *et al.*, 2010 ▶). Moreover, the achieved structural anisotropy is found to become more pronounced if the speed of withdrawal of the sample out of the solution is low (Cho *et al.*, 2006 ▶; Surin *et al.*, 2006 ▶; Valentini *et al.*, 2009 ▶). Dip coating has been employed to solution processable heteroacenes with fused thiophene units, which are highly attractive for OFET applications (De Luca *et al.*, 2011 ▶). Here processing by dip coating has induced an edge-on arrangement towards the surface, with highly ordered structures and long-range orientation of oligomers (Aasmundtveit *et al.*, 2000 ▶; Cho *et al.*, 2006 ▶; De Luca *et al.*, 2011 ▶; Wang *et al.*, 2004 ▶).

Despite the effort made in recent years to apply the dip-coating technique to small molecules and oligomers (Hao *et al.*, 2007 ▶; Sandberg *et al.*, 2002 ▶; Wang *et al.*, 2004 ▶), little is known about the mechanism of conjugated polymer re-orientation with respect to the dipping direction and its relation to field-effect mobilities. Only a few studies have been specifically focused on these matters (Cho *et al.*, 2006 ▶; Hao *et al.*, 2007 ▶; Sandberg *et al.*, 2002 ▶; Wang *et al.*, 2004 ▶) and no consensus has been established yet.

In this paper we demonstrate the ability to achieve chain orientation in regioregular P3HT thin-film transistors by using a dip-coating technique. Improved device performance can be realized in OFET devices by controlling the withdrawal speed and the dipping direction with respect to conducting polymer channels. Both structural and transport properties can be additionally enhanced after thermal treatment.

## 2. Experimental

Regioregular P3HT, *M*_w_ = 44 900 g mol^−1^ and polydispersity index = 1.47, was synthesized at the University of Wuppertal, Germany, and in solution in CHCl_3_ with concentrations of 0.5 and 1.0 mg ml^−1^. For structural characterization, glass substrates (20 × 20 mm) were dipped into a P3HT solution and then withdrawn vertically with controlled speed. Bottom contact OFETs were fabricated by dip coating on patterned testbeds (15 × 15 mm) purchased from Fraunhofer IPMS, Dresden, Germany, allowing 16 OFETs to be obtained from one chip (15 × 15 mm) with different channel lengths of 2.5, 5, 10 and 20 µm at a fixed channel width of 10 mm. Conducting channel lengths of 10 and 20 µm were used for mobility measurements in a saturation regime. The testbeds were cleaned with diluted Hellmanex II (Hellma GmbH) and rinsed with 2-propanol, acetone and distilled water. The mobility measurements were carried out under ambient conditions and remeasured after annealing at a temperature of 453 K for 1 h under N_2_ atmosphere. The X-ray diffraction analysis was performed under grazing-incidence geometry paying particular attention to the in-plane (GID) and out-of-plane (GOD) directions. X-ray measurements were conducted at BL9, DELTA, Dortmund Synchrotron (λ = 0.81 Å), and with a laboratory source (high-resolution Seifert XRD 3003 PTS, λ = 1.54 Å) for thin films with concentrations of 0.5 and 1.0 mg ml^−1^, respectively. At DELTA, a two-dimensional image-plate detector (marCCD) was kept 450 mm apart from the sample with 10 min exposure time for all samples. The incident angle was fixed slightly above the critical angle of the P3HT film (0.11 and 0.2° for the DELTA synchrotron and the laboratory source, respectively) and below the critical angle of the substrate to maximize scattering from the polymer film. *In situ* annealing was conducted at the laboratory X-ray source using an Anton Paar DHS 900 heating stage.

## 3. Results

### 3.1. Structural and transport properties depending on dipping direction

**Figure 1:**
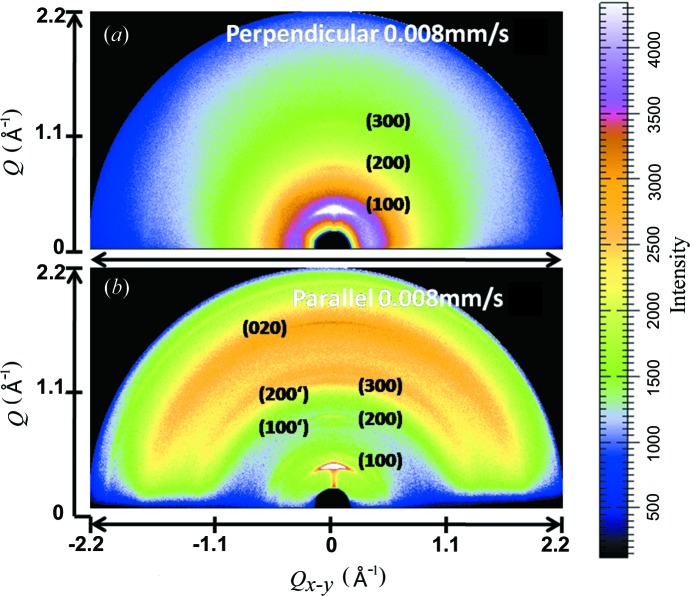
Grazing-incidence two-dimensional patterns in the directions perpendicular (*a*) and parallel (*b*) to the dipping direction.

**Figure 2:**
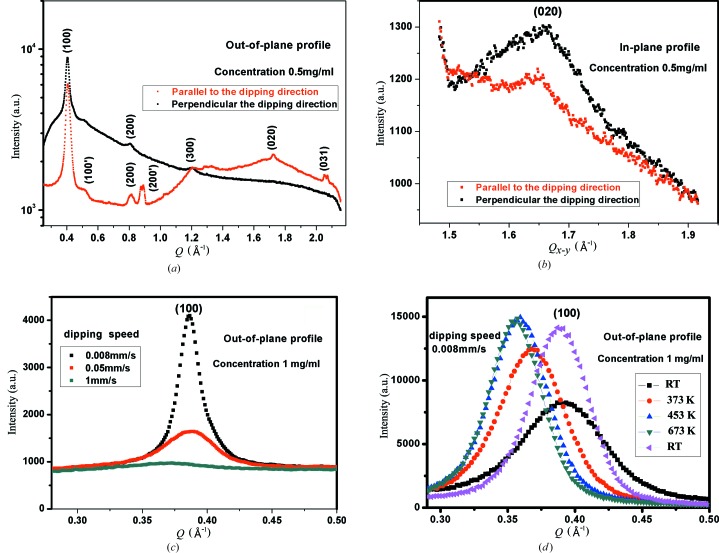
Out-of-plane (*a*) and in-plane (*b*) line profiles perpendicular and parallel to the dipping direction, (*c*) out-of-plane profile of the 100 peaks for dip-coated films with three withdrawal speeds of 1, 0.05 and 0.008 mm s^−1^, and (*d*) *in situ* annealing of the 100 peak for the dip-coated sample with 0.008 mm s^−1^ withdrawal speed.

Two-dimensional patterns of the dip-coated films with a withdrawal speed of 0.008 mm s^−1^ taken perpendicular and parallel to the dipping direction are shown in Fig. 1 ▶ (*Q* and *Q_x-y_* correspond to GOD and GID directions, respectively). The diffraction pattern taken perpendicular to the dipping direction (Fig. 1 ▶ *a*) displays a typical series of *h*00 intense scattering spots. These relatively sharp *h*00 spots along *Q* referring to the domination of edge-on oriented crystallites have been described elsewhere (Joshi *et al.*, 2008 ▶, 2009 ▶). In contrast, the two-dimensional pattern taken parallel to the dipping direction (Fig. 1 ▶ *b*) reveals numerous but less intense *h*00 reflections. In order to compare the diffraction features, line profiles were extracted from the corresponding two-dimensional patterns. The out-of-plane line profiles taken from two-dimensional patterns (Fig. 1 ▶) perpendicular and parallel to the dipping direction are shown in Fig. 2 ▶(*a*). Following previous structural investigations of P3HT films (Joshi *et al.*, 2009 ▶; Sirringhaus *et al.*, 1999 ▶; Kayunkid *et al.*, 2010 ▶), the *h*00 peaks are related to lamellar ordering separated by alkyl side chains. In addition to *h*00 peaks the line profile taken parallel to the dipping direction shows a second polymorph of the *h*00′ series [gray curve (red in the electronic version of the journal), Fig. 2 ▶(*a*)]. The 100′ peak is centered at *Q* = 0.52 Å^−1^, whereas 100 appears at *Q* = 0.40 Å^−1^, corresponding to an interplanar distance of *d* = 11.8 Å^−1^ compared with the main form of 15. 7 Å^−1^. The appearance of higher-order *h*00 peaks and the second form indexed by *h*00′ gives evidence that the structure in the parallel direction consists of two different crystallographic cells. The refined structure has a close agreement with the values reported by Joshi *et al.* (2008 ▶) for thin films and powder P3HT samples (Zen *et al.*, 2006 ▶).

Fig. 2 ▶(*b*) shows line profiles taken from two-dimensional patterns (Fig. 1 ▶) perpendicular and parallel to the dipping direction along the in-plane direction in the vicinity of the 020 peak. In both cases this peak is centered at *Q* = 1.65 Å^−1^, corresponding to a π–π distance of 3.80 Å. This peak is more intense for the perpendicular direction [black curve, Fig. 2 ▶(*b*)] than for the parallel one. Fig. 2 ▶(*c*) shows the 100 peak intensity as a function of the dipping speed, for speeds of 1, 0.05 and 0.008 mm s^−1^. There is a clear dependence on dip-coating speed: the peak intensity increases as the withdrawal speed decreases. This structural enhancement highlights that the variation of the intensity of the (100) planes strongly depends on the interplay between the crystallization rate of the P3HT chains and the evaporation rate of the solvent, since the quality of the dip-coated P3HT films relies on the solvent evaporation rate (Surin *et al.*, 2006 ▶).

### 3.2. Thermal annealing

The enhancement in ordering of the dip-coated P3HT film by the thermal annealing process is shown in Fig. 2 ▶(*d*). Starting from room temperature (RT), the 100 peak intensity (measured in the out-of-plane direction) increases gradually until 453 K. A further increase in temperature up to 493 K shows only marginal changes of the 100 peak. The overall gain in intensity is accompanied by the shift of the peak maximum to smaller *Q* values, indicating lattice expansion similar to the previous findings (Joshi *et al.*, 2008 ▶). Upon cooling down to RT the 100 peak shifts back to the original *Q* position, but becomes more intense and narrower in peak width (FWHM) compared with the initial film. The intensity increases by a factor of two between the initial and post-annealed films (Fig. 2 ▶ *d*). The *h*00 intense and narrow peaks refer to an interplanar distance of 15.7 Å associated with the ordering of hexyl side chains. Similar *d* spacing was observed for spin-cast P3HT prepared from CHCl_3_ solution (Zen *et al.*, 2004 ▶).

### 3.3. Electrical characterization

Bottom gate and source/drain OFETs were prepared using three different withdrawal speeds (1.0, 0.8, 0.5 mm s^−1^) of the SiO_2_ substrate from P3HT/chloroform solution (0.5 mg ml^−1^) at room temperature. OFETs were subsequently annealed in a glass oven at 453 K in a nitrogen atmosphere. Transfer and output measurements are necessary to extract the performance parameters of OFETs, such as threshold voltage, the field-effect mobility and on/off current ratio. The transfer and output curves have shown respectable transistor behavior as the pulling (withdrawal) speed decreases. The variation of the field-effect mobility as a function of withdrawal speed was monitored, and the results revealed that the mobility increased with lowering withdrawal speed (see Table 1 ▶).

The influence of the dipping speed on OFET performance is shown in Table 1 ▶. For the highest dipping speed of 1 mm s^−1^ there is no pronounced difference in mobility between the parallel and perpendicular dipping directions. A further decrease of the withdrawal speed is accompanied by a substantial increase of OFET mobility for films with the conductive channels perpendicular to the dipping direction.

## 4. Discussion

The results obtained suggest that there is a clear correlation between the structural and transport properties of the P3HT thin films: the better OFET performance of samples prepared at lower speed is explained by the larger number of crystallites ordered in the preferred edge-on configuration. Altogether these results highlight that the microscopic morphology is sensitively dependent on the interplay between the crystallization rates of the regioregular P3HT chains and the evaporation rate of the solvent (chloroform), which can be tuned by the dipping speed. A further improvement in the mobility of OFETs was achieved by dipping in a direction perpendicular to the orientation of the conducting polymer channels. Moreover, from Table 1 ▶ it is apparent that the film dipped perpendicularly to a conducting channel, and annealed afterwards, provides higher OFET mobility (1.81 × 10^−3^ cm^2^ V^−1^ s^−1^), which differs by nearly one order of magnitude from the values obtained in the parallel direction (4.65 × 10^−4^ cm^2^ V^−1^ s^−1^). X-ray studies demonstrate that, despite the presence of multiple reflections in the diffraction pattern in the parallel direction where the second polymorph with *h*00′ reflections is clearly visible, the strongest π–π conjugation takes place for dipping in the perpendicular direction. Therefore the enhancement in the edge-on crystalline orientation and improvement of π–π conjugation are mainly responsible for the increased mobility upon decreasing the withdrawal speed. For OFETs with conducting channels perpendicular to the dipping direction the mobilities are revealed to be improved by one order of magnitude. It should be noted that, owing to the weak scattering signal with increased dipping speed (Fig. 2 ▶ *c*), structural studies were focused on the lower dipping speed case. Our findings support that a dip-coating technique can be an effective low-cost fabrication method for large-scale organic devices.

## Figures and Tables

**Table 1:** P3HT OFET mobilities as a function of withdrawal speed and temperature for conductive polymer channels perpendicular and parallel to the dipping direction

Dipping direction	Speed (mm s^−1^)			Temperature
	0.5	0.8	1.0	
Parallel	2.83 × 10^−4^	4.54 × 10^−5^	1.85 × 10^−5^	Room
Perpendicular	6.42 × 10^−4^	2.67 × 10^−4^	3.41 × 10^−6^	Room
Parallel	4.65 × 10^−4^	5.38 × 10^−5^	3.11 × 10^−5^	453 K
Perpendicular	1.81 × 10^−3^	1.49 × 10^−3^	4.04 × 10^−5^	453 K
